# Biology-inspired engineering for circular bioeconomy systems

**DOI:** 10.1186/s13036-025-00527-7

**Published:** 2025-06-19

**Authors:** Brahm P. Verma, James W. Jones

**Affiliations:** 1 College of Agricultural and Environmental Sciences, Biological and Agricultural Engineering, and College of Engineering, Institute of Biological Engineering, University of Georgia, 1120 Lakewood Manor Drive, Athens, GA 30606 USA; 2 Department of Agricultural and Biological Engineering, U.S. National Academy of Engineering, University of Florida, Gainesville, FL 32611 USA

## Abstract

This article presents perspectives on the need to transition from the current unsustainable consumptive fossil-based linear (take-make-use-dispose) systems that produces huge quantities of wastes, pollutes land, water and air, and contributes to climate change to sustainable bio-based circular (take-make-use-decay-reuse) systems. In the article, the word ‘fossil’ refers to all forms of mined carbon and minerals from the Earth, including water from aquafers, which cannot be replenished at the rate that will maintain their capacity to provide for the future. The natural world through its many circular systems uses energy and renewable resources to perform functions that produce zero waste. One organism’s waste becomes another organism’s food, material, and energy, forming a circular loop (take-make-use-decay-reuse). Over the past 4 years, deliberate engagements with leaders of multiple disciplines and stakeholders resulted in conclusions that the problems of the complex biologically active systems (biosystems) that are intertwined with natural systems and socio-economic systems can only be addressed by having a robust culture of convergent science and engineering and systems-thinking for transitioning from linear fossil-based to circular bioeconomy systems. We present the need and propose forming a multidisciplinary professional society alliance to promote and support networks of multidisciplinary teams to address problems of complex, intertwined bio-natural-socio-economic systems of systems. This article proposes that the Institute of Biological Engineering (IBE), a society whose primary objective is to “to apply biology-inspired engineering principles to design systems to improve the quality of the human condition”, and inculcates a culture of convergent science and engineering that has members representing expertise of multiple science and engineering discipline, is potentially an excellent candidate to play a pivotal role in designing innovative solutions for advancing sustainable circular bioeconomy systems.

The current U.S. economy is predominantly linear and unsustainable in that it produces huge qualities of wastes, contributes to climate change, and uses non-renewable fossil-based resources. The bioeconomy is a smaller but an important part of the current economy that uses renewable biological resources (like crops, forests, animals, and microorganisms) to produce materials and services. The bioeconomy refers to economic activities derived from the life sciences, particularly in the areas of biotechnology and biomanufacturing, and includes industries, products, services, and the workforce [1].

An essential societal goal is to transition the current economy into a more circular bioeconomy system that uses renewable biological materials and adopts key features of nature’s cyclical processes where biomaterial “wastes” are used as biological resource feedstocks for synthesizing new materials. Figure 1 depicts the current mostly linear economy and its transition into a more circular and larger future economy in which the use of renewable biological resources in economy (bioeconomy) will reduce reliance on fossil resources (mined carbon and minerals from the Earth) and is the dominant component of the economy.

**Fig. 1:**
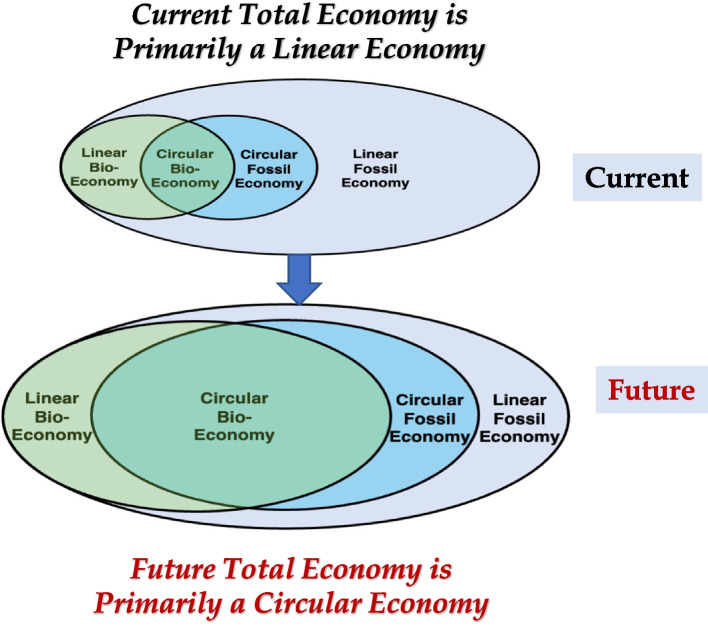
Upper Figure illustrates proportions of linear, circular, fossil-based, and biobased economy systems in the current economy. The lower Figure illustrates the envisioned transformed future total economy in which the circular bioeconomy is the dominant component of the whole economy and larger than in the current economy

This article describes the challenges of transitioning the economy from linear to circular while simultaneously replacing non-renewable fossil-carbon inputs with renewable biological materials. We present an overall perspective on the importance of convergence (that is, integration of knowledge, tools, skills and ways of thinking beyond a single discipline) to problem solving, and the importance of biological engineering in creating innovations for complex irreducible bio-systems shown in Fig. 2 [2].

**Fig. 2:**
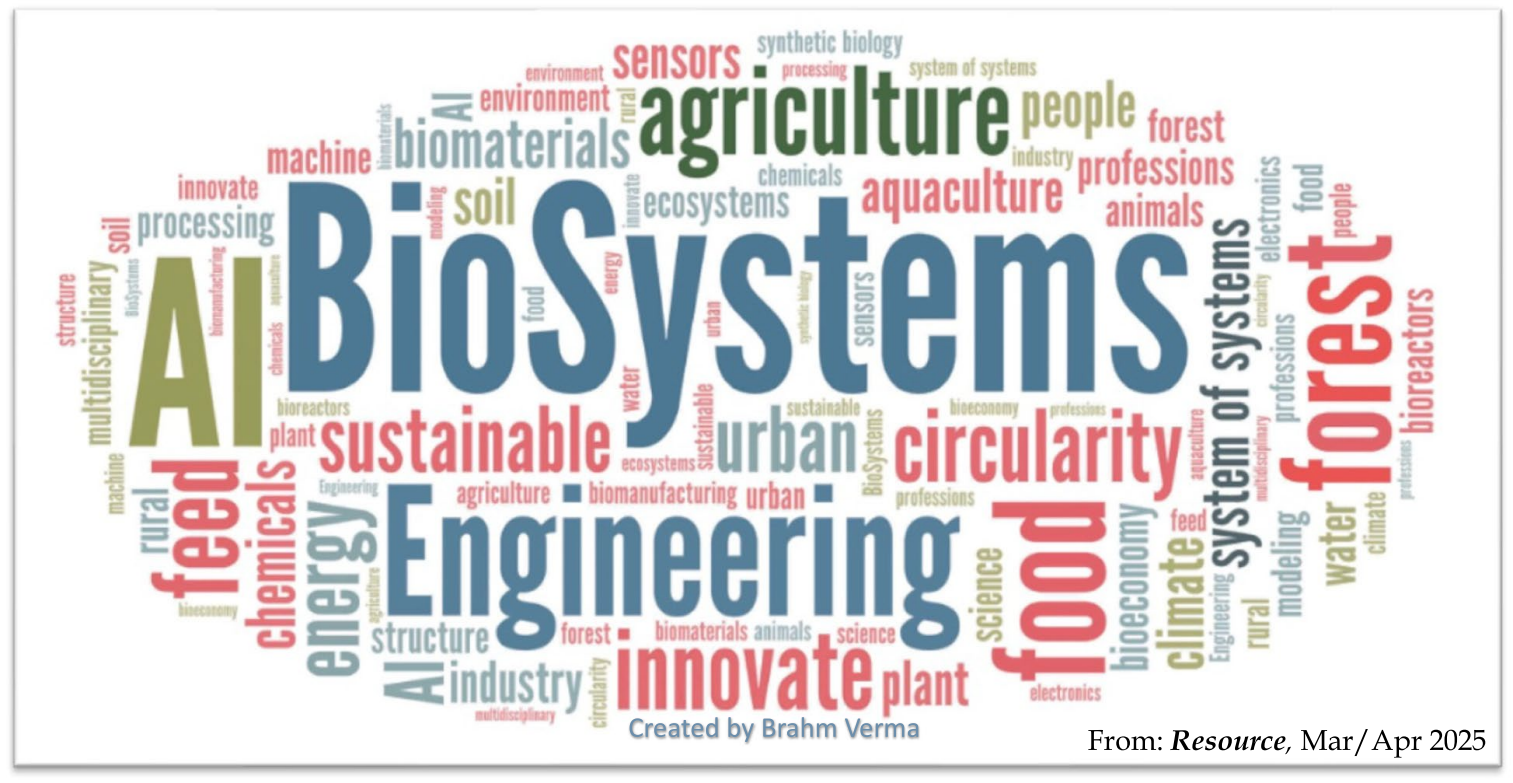
Graphically represents that biosystems. They include systems that use natural resources of land, air, water and energy to perform functions and synthesized products by modulating biological activities

Linear economy systems *take-make-use-dispose* resources, where disposed resources are largely wasted and pollute our soils, water, air, and adversely affect the Earth’s ecosystems by depleting capacity to provide essential resources needed for the future economy systems. In a linear economy, the use of energy, materials, and labor is optimized primarily for performing desired functions within each constituent system. The embedded resources contained in after-use-materials and byproducts, in a large part, are irretrievably discarded as waste and thus not accessible for beneficial or profitable reuse. The cost of lost resources is externalized and not accounted for, giving a false accounting of economic benefits of the current system.

In the past hundred years, the U.S. food and agricultural biosystems (FABS) quadrupled the supply of food and fiber [3] and have made impressive contributions by nearly eliminating natural famines and reducing poverty to reach the lowest level globally in the recorded history. It constitutes nearly a quarter of the current US economy and workforce. However, because they are predominantly linear systems, FABS are also significant sources of emissions and pollutants contributing to climate change (Fig. 3). Actions to solve visible problems, such as losses due to diseases and pests, within each constituent systems largely ignore unintended consequences and serious resource issues.

**Fig. 3:**
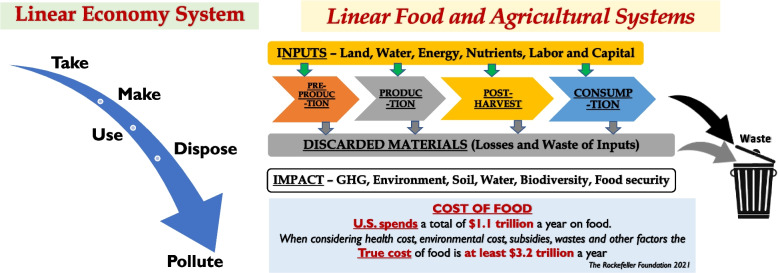
Left Figure shows the flow of energy and materials in a linear economy system that discards resources and produces waste. Right Figure – shows linear bioeconomy (food and agricultural) systems in which nearly two-third value of the input resources of the entire value-chain is lost when considering health cost, subsidies, and waste costing nearly $2.1 trillion a year

The Food and Agricultural Organization (FAO) of the United Nations (UN) [4] estimated that the global food and agriculture sector contributes about 31% of human-caused greenhouse gas emissions (21% of all world CO2, 53% of all methane, and 78% of all NOx), and about one-third of all food grown in the world is wasted. A 2021 Rockefeller Foundation [5] study reported that, when considering health cost, environmental cost, subsidies, waste and other factors, the additional cost to the U.S. economy is at least $2.1 trillion a year, totaling to $3.2 trillion.

The Earth’s climate is changing at an unprecedented and alarming rate. There is a strong consensus that much of the current unprecedented changes are, at least in part, a result of human activities driven by industrialization and enhanced humans’ capacity to mine resources at alarming rates, and ability to deploy advanced technologies for human needs. Industrialization and biosystems demand mined resources in their own linear (non-circular) systems. These changes have primarily propelled the growth of the linear economy that is heavily dependent on mined resources. If we focus on circularity and reduction of demand for mined resources, then fossil demand will consequently be lowered.

Jones [6]) reported that the U.S. food, agriculture and other biosystems are at a crossroad as they prepare to meeting such daunting challenges for 2050 as:

- Increase food supply by nearly 70% to meet demands of the global population expected to reach 10.5 billion
- Improve resource use efficiency when Earth’s per capita available resources (fresh water, arable land, fossil-fuel based energy, and chemicals) will be reduced by over 30%
- Develop strategies and systems of transportation and distribution for an urbanized global population (with nearly 75% people living in urban areas)
- Increase availability of biomass to provide renewable feedstocks that replace fossil resources for biomanufacturing, in addition to providing needed food, feed, fiber, and bioenergy
- Drastically reduce GHG emissions, nutrient losses, and pollution, and increase eco-services and sequestration of carbon in soil.
- Build resiliency to prepare for unexpected events, and climate change

The natural world, through its many circular systems, use energy and materials to perform nature’s functions while dynamically interacting to changing conditions and producing no waste. Morton and Shea [7] present this concept as “Circles of Life” represented in Fig. 4.

**Fig. 4:**
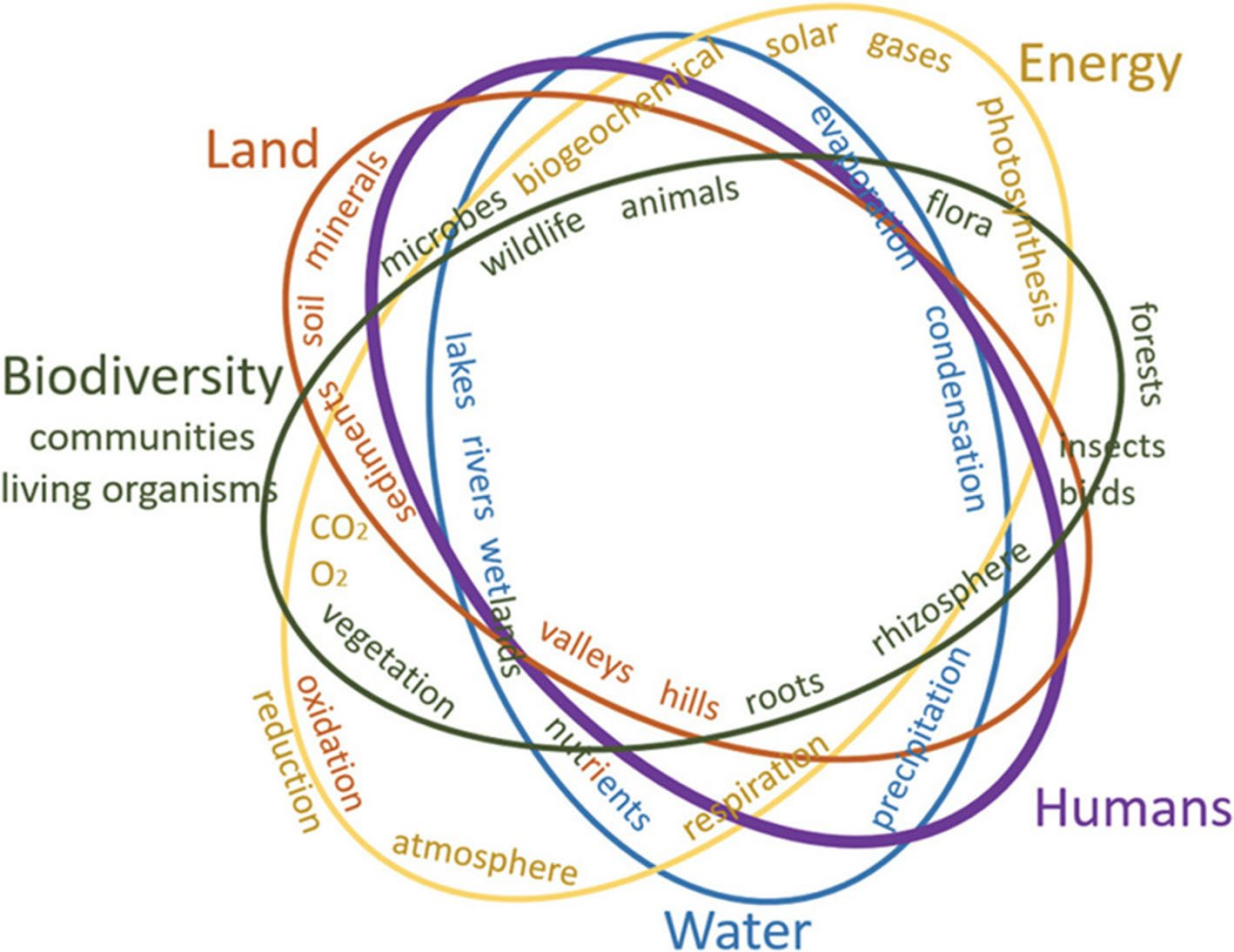
Circles of life in the natural world include many dynamic interwoven processes over space and time [7]

Adopting principles of circularity will require developing knowledge and techniques for maintaining embedded resources within economic systems; characterizing, capturing, and quantifying ‘wastes’; developing methods for extracting and processing resources that can be reused or recycled or upcycle as feedstock for biomanufacturing products; and synthetizing materials and chemicals for human need. Figure 5 illustrates an overall concept of circular economy system.

**Fig. 5:**
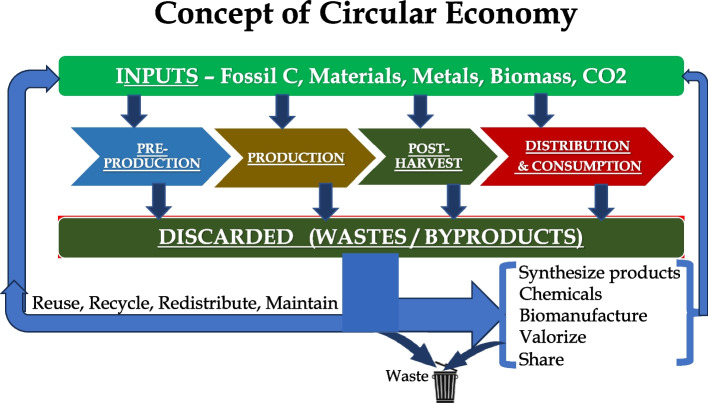
The illustration shows the concept of circular economy in which resources embedded in discarded materails (waste) are captured and recycled or reused to synthesize new products, chemicals and other valueable products, and waste is reduced

Whereas the current economy is largely a product of physical processes and devices, Nature’s tools are living organisms, biochemical processes, and molecular ‘machines’. They optimize the use with system’s capacity to replenish resources to perform functions within a constituent system. However, functions are selected in the context of consequences they may have on other interwoven constituent systems to avoid unexpected results affecting the whole system. Furthermore, they keep products and materials in use as long as possible, and after-use-materials and byproducts become the input resources of other systems producing zero waste. That is, one organism’s ‘waste’ becomes another organism’s food, nutrient, and energy, thus forming a circular loop of *take-make-use-decay-reuse*. A recent McKinsey Global Institute study [8] reported that “as much as 60 percent of the physical inputs to the global economy could, in principle, be produced biologically.”

Bioeconomy systems are complex irreducible systems. The entire bioeconomy value-chains are interwoven with Nature’s ecosystems and socio-economic systems in which commerce is affected by behaviors of people in making policy and economic transactions of goods and services. These three systems form complex relationships (Fig. 6) to produce biomass on land, in water, in laboratories or elsewhere (agriculture, forest, marine, aquaculture, and bioreactors); synthesize them into new products, chemicals, and materials for human needs (e.g., food, feed, fiber, fuel, chemicals, and more); and provide ecosystem services to sustain natural resources.

**Fig. 6:**
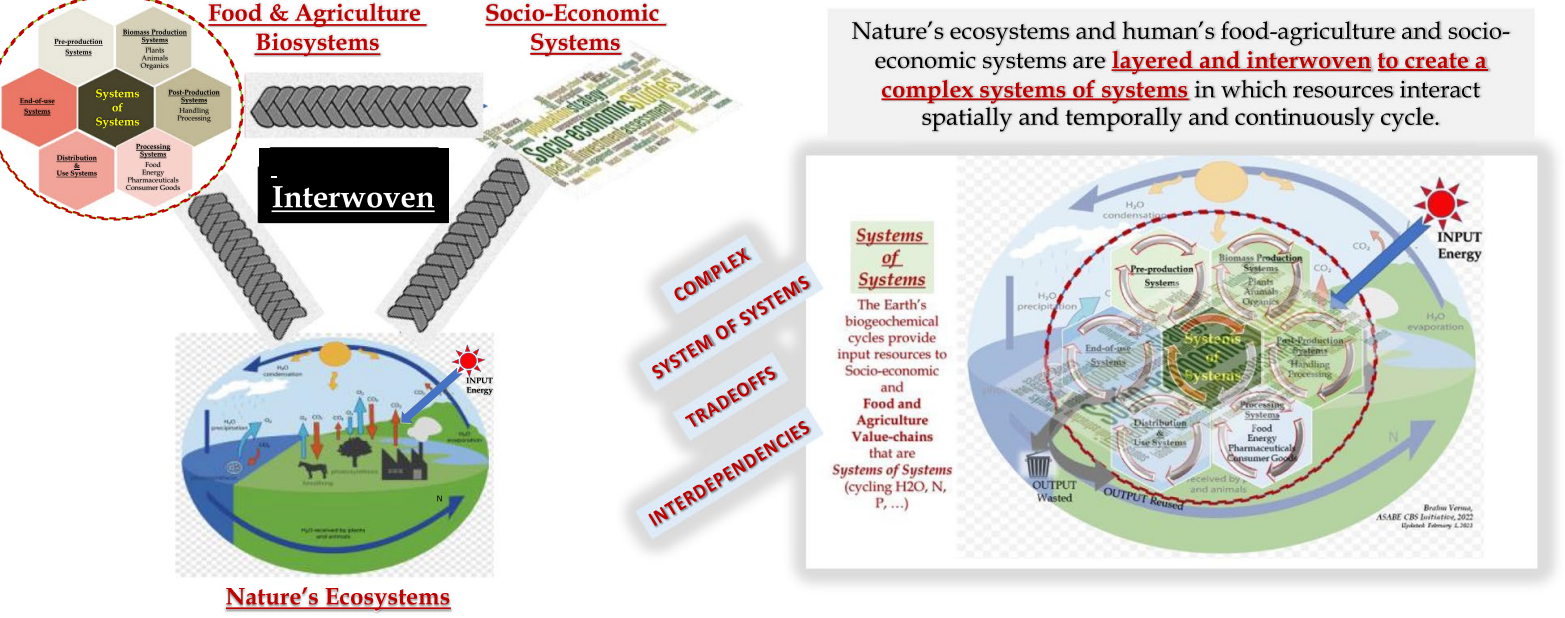
Figure on the left shows the three (food and agriculture, natural and socio-economic) systems are interwoven. Figure on the right shows that these three systems are also layered and networked. The two Figures together depict complex systems of systems in which any actions in any part of the system may result in unintended consequences to the performance of the whole system

A 2020 National Academies of Science, Engineering, and Medicine report [9] identified four drivers of the U.S. bioeconomy: life sciences, biotechnology, computer and information sciences, and engineering shown in Fig. 7. We identify behavioral, social and political sciences as also being important drivers of bioeconomy shown as the fifth wheel in brown color in Fig. 7.

**Fig. 7:**
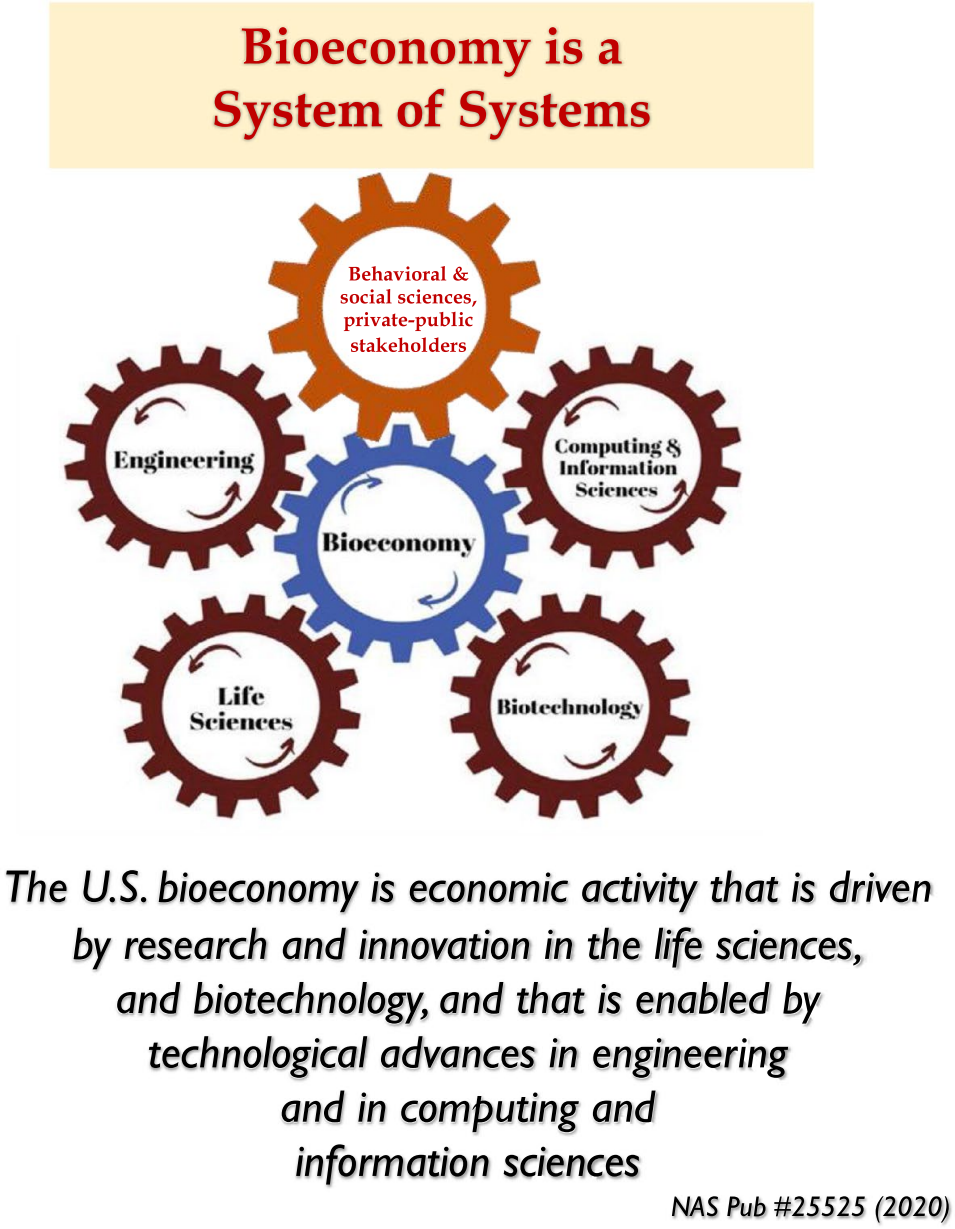
The four brown gears representing life sciences, biotechnology, computing and information sciences and engineering drive the blue bioeconomy gear as presented in the National Academies report (2020). We add behavioral, social, and political sciences and stakeholders as another driver of the bioeconomy shown by the fifth gear in brown color. Individuals and society behavior are integral drivers of the bioeconomy

Morton and Shea [7] observed that when systems relationships are ignored, decisions that address the visible problem of constituent systems, such as selecting pesticides and application methods without considering impacts on the Earth ecosystems, may create a myriad of unintended consequences and serious resource issues. Understanding and conceptualizing solutions in the context of multi-directional interconnections in complex systems require systems thinking. It also requires a simultaneous consideration of consequences of any one or more interacting subsystems on the behavior of the whole system. To develop knowledge that provides a deeper understanding of complexities of interwoven systems will require convergent science and engineering that transcends disciplinary boundaries and adopts systems thinking. Solutions for identified problems of each component system will need to be thoroughly vetted to predict behavior of the whole system and avoid unintended consequences. Reductionist approaches of finding disciplinary-based solutions to recognized problems in constituent systems will not provide desired system-level outcomes; in fact, in many cases system-level outcomes could be detrimental.

These challenges and potentials of circular systems for addressing them came in focus at the 2019 Annual International Meeting of the ASABE. The keynote speech “*From Here to Sustainability”* by Joel Makover, President and CEO of Greenbiz, led to thoughtful deliberations among ASABE members and leadership groups. In 2020, the American Society of Agricultural and Biological Engineers (ASABE) president committed the society to lead a national initiative *Transformation of Food and Agricultural Systems (FAS) to Circular Systems* [10] and appointed a roundtable of 27 ASABE members to envision several food and agriculture circular systems. Another brainstorming session with a 37-member focus group, which included representative of 14 disciplines and stakeholder groups, identified a need and core objectives for a multi-society partnership for developing circular systems. ASABE then appointed a Circular Bioeconomy Systems (CBS) Task Force (see Appendix 1) that developed and recommended using the following five principles of circularity for designing a circular bioeconomy system:

1. Increase use efficiency that reduces the need for input resources,
2. Design out waste and pollution towards the goal of achieving zero waste,
3. Keep products and materials in use within the system as long as possible,
4. Regenerate natural systems, and
5. Provide economic benefit as an incentive for adoption into the general economy.

These principles were adopted by adding the first and the fifth principles to the three principles recommended by Ellen Macarthur Foundation [11]. A design of circular bioeconomy system should strive to meet all five principles of circularity.

Figure 8 shows a conceptual circular bioeconomy system in which input biological materials produces products as well as waste at every stage of the value-chain. The capacity to produce a wide variety of products (food, fuels, pharmaceutics, cosmetics, building materials, chemicals, and more) and ways to use waste (especially biological waste) to ‘manufacture’ economically profitable products and services by some means toward achieving the zero-waste goal, are limited by our imagination and capacity to innovate.

**Fig. 8:**
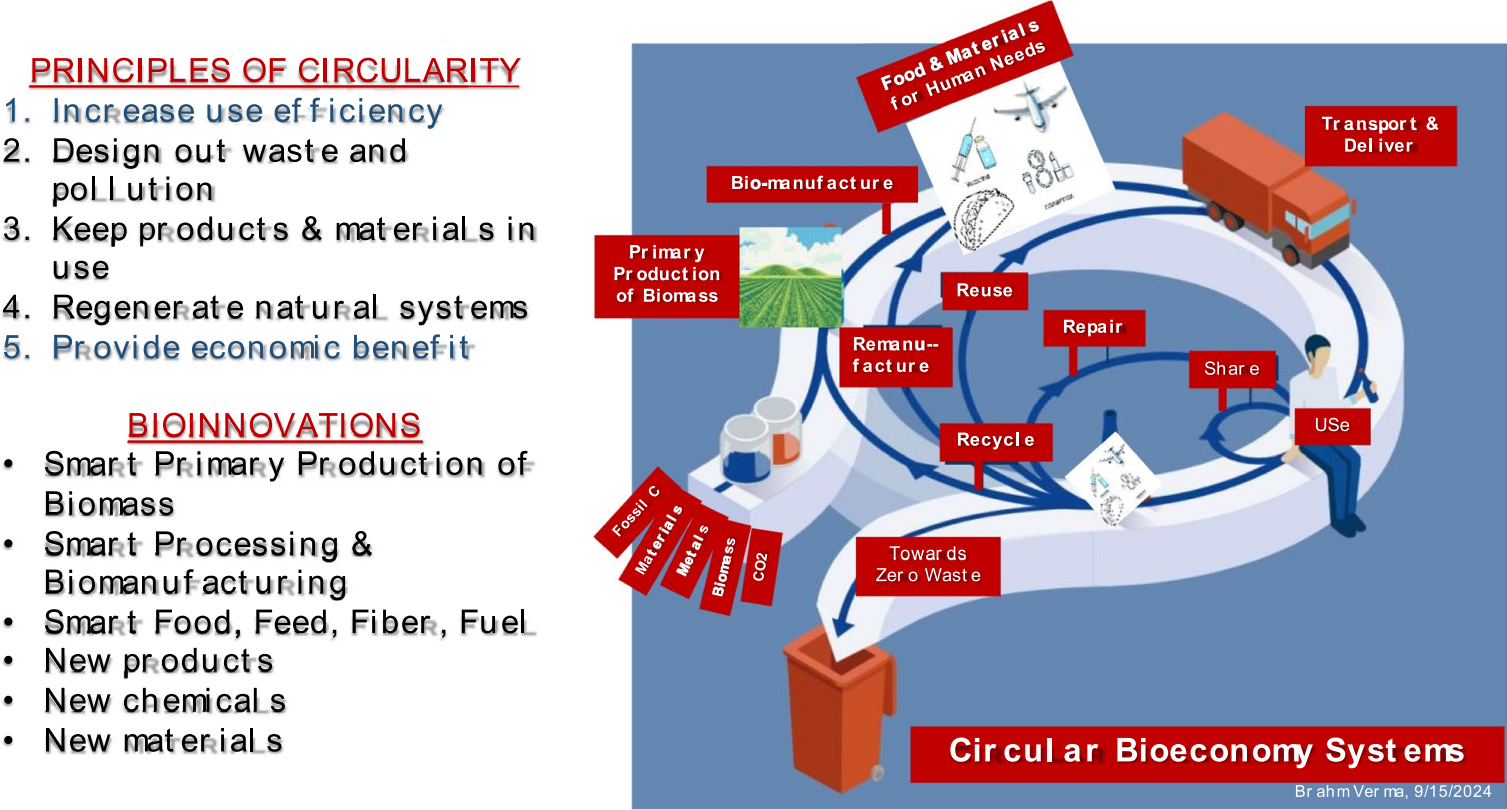
The Figure illustrates the flow of resources in a circular bioeconomy with products and waste at all stages of the value-chain. The Figure shows biological materials as input resources for synthesizing a vast variety of products (food, pharmaceutics, cosmetics, fuel, and others yet to imagine), recapturing resources by various techniques, and using them for other useful purposes to move toward producing zero waste. To achieve circularity illustrated in the Figure, one must use all five principles of circularity in conceptualizing circular systems (listed on the top left) and apply operating principles of *New Biology* [12, 13] to innovate ‘smart’ solutions and produce new products, chemicals and materials (listed in the lower left)

The study of the living world has always been by combining observations from biology, geology, and physics. In 1939, Compton and Bunker [12] observed that biology is increasingly becoming more analytical and called it the ‘Modern Biology’. During the same time as advances in the development of engineering sciences based on physics and chemistry were changing the practice of engineering, they proposed an engineering curriculum based on the ‘modern’ biology. They observed that “within this concept lies ample scope for every activity from instrumentation to theory, so long as the major objective is the marshalling of all available resources to aid biology for the benefit of humanity.”

A U.S. National Academies committee [13] reported that the integration of the subdisciplines of biology and with greater integration of it with the physical and computational sciences, mathematics, and engineering, a “New Biology” is emerging in order to tackle system level questions in quantitative ways. The deeper understanding of biological systems, that is, their operating principles and biochemical processes, presents the promise of predicting and modulating behaviors, to develop biology-based systems-level solutions. The Committee also identified food, environment, energy, and health as the four broad societal challenges that could be tackled by the New Biology (Fig. 9a). These challenges are implicit in the challenges to transition to circular systems.

**Fig. 9:**
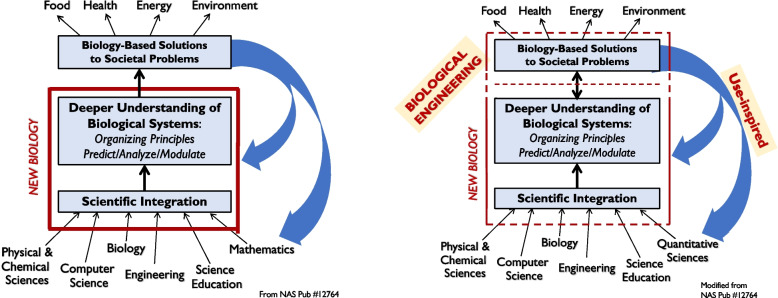
**a** (Left). Shows the integration within biology and with physical, chemical and information sciences and engineering (bottom block) is making possible to understand operating principles of living systems, and making it possible to modulate and predict activities of biological systems in ever greater details (middle block), and use this knowledge to design solutions (upper block) for societal problems. (From NAP 12764). **b** (Right). Modified Figure identifies the evolution of the engineering science based on biology (biological engineering) for designing use-inspired solutions that inform the sciences and contribute to deeper understanding of biological systems

In Fig. 9b, we show three observations:

1. the upper block (Biology- based Solutions to Societal Problems) is labeled biological engineering in the model conceptualized by Compton and Bunker [12] in 1939,
2. the one-directional arrow between the middle block and upper block is replaced with two-directional arrow to show that biological engineering is an enabling science that contributes to deeper understanding of biological systems, and
3. the use-inspired engineering contributes to advances in all fields of sciences disclosing the underlying unifying principles of apparently unrelated branches through which comes convergence.

Here is one example that demonstrates the untapped possibilities. Living organisms and nature’s processes provide unlimited opportunities to develop circular systems. Meyer and Schaffner [14] described that fungi have incredibly diverse metabolic capabilities and they are everywhere in the biosphere. Using biocatalysts, they decompose wood into raw materials and synthesize (recombine and reassemble) them into new products. An interdisciplinary group of biotechnologists, artists, and architects MY-CO-X created a 20 square meter inhabitable structure MY-CO-SPACE [15] made of fungi and wood. The fungal mycelium was grown in agricultural and forest residues (bark, sawdust, or concrete recycles from demolition waste). They reported that as it grows, the particles get compressed resulting in forming hard composite material. The network of fungi filaments grows increasingly dense, acting like mortar. The natural world’s abundant microorganisms performing complex tasks provide unlimited opportunities to convert and use current waste and biomanufacture products to create a sustainable circular bioeconomy system that serves the societal needs.

To accelerate the pace of progress, we need local, regional, and national (even international) networks of multidisciplinary teams to engineer biological systems, and use the five principles of circularity to design practical sustainable circular systems producing near zero waste.

Academic institutions, industrial and national laboratories play an important role by forming multidisciplinary research and education teams within their organized centers and institutes. They form teams primarily by recruiting talent from diverse disciplines within their institutions to focus societal problems aligned with institutional strategic priorities. On the other hand, disciplinary professional societies primarily support the single disciplines for which they are named, uniquely influence and contribute to the growth of the discipline by organizing forums (meetings, conferences), promote activities to develop professional excellence, raise public awareness, publish high-impact single-discipline focused journals, and in other ways formulate disciplinary worldview and priorities for fulfilling overall societal needs.

The Institute of Medicine consensus study [16] and National Academies workshop [17] identified the importance of interdisciplinary efforts and requirements for fostering a culture of convergence for addressing complex system problems. These reports provide insights on how partnering disciplinary societies could play critical roles in the growth of a multidisciplinary culture, publish results in high-impact journals, address concerns and achievements of their members, and raise public awareness.

The CBS ASABE Task Force formed a 26-member multidisciplinary work group (see Appendix 2) to draft features and mechanisms that will bring together professional societies to build partnerships and form an alliance to create innovative and sustainable circular bioeconomy systems. The work group included leaders of 8 professional societies (including five members of the U.S. National Academies), foundation presidents, and stakeholder representatives. It developed a consensus model of multidisciplinary professional society alliance for circular bioeconomy systems shown in Fig. 10 [18].

**Fig. 10:**
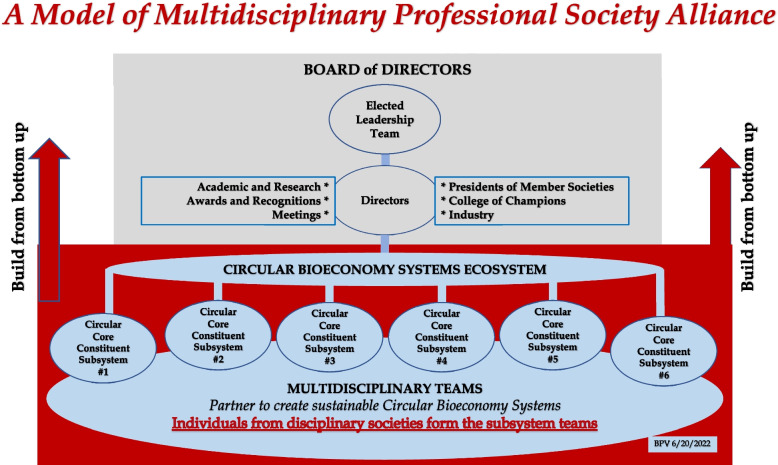
A model of multidisciplinary professional society formed as a *Joint Venture* by building alliances with the participating disciplinary professional societies. The top block (in grey) illustrates the operating and executive functions, and the bottom block (in red) shows several organized and self-organized multidisciplinary working teams formed by members of institutions, agencies, foundations, and stakeholders to perform tasks for innovating system-level solutions for advancing circular bioeconomy systems. The two red arrows pointing upwards, signify that the formation of the alliance should begin from bottom, that is, first from several multidisciplinary teams to innovate solutions for relatively easier problems. Their successes and experiences will be motivations to accept or refine the operating and executive structure [17]

The model alliance is structured to be a *Joint Venture* among societies to benefit all partnering disciplinary societies as they work together to form multidisciplinary teams to develop knowledge and innovative solutions for problems of constituent systems. The alliance model provides opportunities for members to self-organize, and in general, learn to address unexpected emergence of properties/behaviors of complex systems. The primary functions of the alliance are to identify priority problems and organize working teams that promote universities, research laboratories, and industry to organize programs and funding agencies to provide resources for finding solutions. In a way, the alliance is simply a multidisciplinary professional society performing similar functions that professional societies generally do.

The Institute of Biological Engineering (IBE) has an important role in the forming multidisciplinary partnerships for advancing circular bioeconomy systems. The primary objectives of IBE are “to apply biology-inspired engineering principles to design systems for improving the quality of the human condition*”,* and to inculcate a culture of convergent science and engineering. Like other professional societies, IBE supports activities for advancing the discipline of biological engineering inspired by the inherently interdisciplinary science of biology (or New Biology). Unlike other professional engineering and scientific societies, the scope of IBE is agnostic to the fields of application. It includes members with expertise in many sub-fields of biology and engineering, who focus on problems of food, environment, health, and energy. Thus, IBE is a natural candidate to engage in and play a central role in the advancement of circular bioeconomy systems, and become a catalyst to organize activities that will lead to the formation of the envisioned multidisciplinary alliance.

In conclusion, application principles of circularity (Fig. 8) by mimicking nature’s *Circles of Life* presents a great potential to make sustainable circular bioeconomy systems a dominant part of the whole economy. CBS shows the promise of meeting the upcoming daunting challenges of increasing biomass for food, feed, fiber, bio-feedstock to replace fossil resources, with fewer available natural resources (land, fresh water and nutrients). Because bioeconomy is complex, intertwined biobased-systems, natural-systems, and socio-economic-systems of systems with the potential of providing ecosystem services, the designing solutions for problems of a constituent system must be in the context of the whole system. This will require scientific integration for developing deep quantifiable understanding of operating principles of biological systems. The advancements of the *New Biology* and its use for developing the engineering science of biology presents new ways to solve societal problems. A multidisciplinary professional society alliance is needed to create an ecosystem for innovating solutions for advancing circular bioeconomy systems. The Institute of Biological Engineering (IBE), a society whose primary objective is to “to apply biology-inspired engineering principles to design systems for improving the quality of the human condition” inculcates a culture of convergent science and engineering. Thus, IBE is an excellent candidate to play a pivotal role in forming a collaborative-multidisciplinary ecosystem for innovating solutions to advance sustainable circular bioeconomy systems.

## Data Availability

No datasets were generated or analysed during the current study.

## Appendix 1

List of Members of the Circular Bioeconomy Systems (CBS) Task Force.

(Appointed by the President of the American Society of Agricultural and Biological Engineers).

**Table:**

Name	Title and Affiliation
1. James (Jim) W. Jones, *Chair*	*Distinguished Professor Emeritus of Agricultural and Biological Engineering, University of Florida; and Member of the National Academy of Engineering*
2. Richard (Rick) Koelsch	*Professor Emeritus of Biological Systems Engineering, University of Nebraska*
3. Edward (Ed) Barnes	*Sr. Director of Agricultural and Environmental Research Division, Cotton Inc*
4. Carolyn Jones	*Bay/Delta Team Agricultural Engineer, USDA—Natural Resource Conservation Service, California*
5. Sudhagar Mani	*Professor of BioChemical Engineering, University of Georgia*
6. Angela Green-Miller	*Associate Professor of Agricultural and Biological Engineering, University of Illinois Urbana-Champion*
7. Lara Moody	*Executive Director, Institute for Feed Education and Research (IFEEDER)*
8. Sue Nokes,	*Acting Associate Provost, University of Kentucky; and Past President of ASABE*
9. Allen Rider	*Retd. President of New Holland North America; Past President of ASABE and ASABE Foundation*
10. W. Joe Sagues	*Assistant Professor of Biological and Agricultural Engineering, North Carolina State University*
11. R. Paul Singh	*Distinguished Professor of Food Engineering Emeritus, University of California, Davis; and Member of the National Academy of Engineering*
12. K.C. Ting	*Professor and Head Emeritus, Agricultural and Biological Engineering, University of Illinois Urbana-Champion*
13. Veronika Vazhnik	*Sustainability Consultant, Governor of Idaho’s Sustainability Office*

## Appendix 2

List of Members of the Working Group for Drafting a Plan for a Professional Society Alliance.

(Appointed by the ASABE CBS Task Force).

**Table:**

Name	Title and Affiliation
1. Brahm P. Verma, *Chair*	*Professor and Associate Director of Engineering Emeritus, University of Georgia; and Founding President of the Institute of Biological Engineering*
2. Barbara Amon	*Sr. Research Scientist**, **ATB, Potsdam Inst., Germany; and President of European Society of Agricultural Engineering*
3. Bruno Basso	*Foundation Prof., Plant, Soil & Microbial Sciences, Michigan State University*
4. Ziynet Boz	*Assistant Professor of Food Engineering; University of Florida*
5. Ximing Cai	*Ben Chie Ven Professor, Civil & Environmental Engineering**, **University of Illinois Urbana-Champion*
6. Geoffrey Dahl	*Distinguished Prof. Dairy Science, University of Florida, and Past President of the American Dairy Science Association *
7. Gal Hochman	*Professor of Agricultural & Applied Economics, Rutgers University*
8. David Jones	*Professor and Head, Biological Systems Engineering, University of Nebraska*
9. James (Jim) W. Jones	*Distinguished Professor Emeritus of Agricultural and Biological Engineering, University of Florida; and Member of the National Academy of Engineering*
10. Carolyn Jones	*Bay/Delta Team Agricultural Engineer, Bay/Delta Team Agricultural Engineer*
11. Madhu Khanna	*Distinguished Professor of Agricultural. & Applied Economics, University of Illinois Urbana-Champion; and Past President of Agricultural & Applied Economics Association*
12. JoAnn Lighty	*Professor of Biochemical Engineering and Dean of College of Engineering, Boise State University*
13. Vara Prasad	*Distinguished Professor of Crop Science, Kansas State University; and* *Past President of Crop Science Society of America*
14. Sharvari Raut	*Scientific Researcher**, **ATB, Potsdam Inst., Germany*
15. John Reid	*Research Professor of Engineering, University of Illinois Urbana-Champion; and Member of the National Academy of Engineering*
16. Allen Rider	*Retd. President of New Holland North America; Past President of ASABE and Past President of the ASABE Foundation*
17. Maury Salz	*Retired President of CLAAS Omaha; Past President of ASABE; and President of the ASABE Foundation*
18. Gretchen Sassenrath	*Professor Soil Science, Kansas State University*
19. Adel Shirmohammadi	*Professor of Agricultural and Biological Engineering, University of Maryland*
20. Ernie Shea	*President, Solution from the Land*
21. Barbara Strum	*Scientific Director, ATB, Potsdam Inst., Germany; and President EuAgrEng*
22. R. Paul Singh	*Distinguished Professor of Food Engineering Emeritus, University of California, Davis; and Member of the National Academy of Engineering*
23. KC Ting	*Professor and Head Emeritus, Agricultural and Biological Engineering, University of Illinois Urbana-Champion*
24. Keith Tinsey	*Dir. of Operations, Black Gold Farms; ASABE President*
25. Lalit Verma	*Professor and Head Agricultural and Biological Engineering**, **University of Arkansas; President of the ASABE Foundation; and Past President of ASABE*
26. David Zilberman	*Distinguished Professor of Agricultural & Resource Economics, University of California, Berkeley; and Member of the National Academy of Science*
Darrin Drollinger, *Ex-Officio*	*Executive Director of ASABE*
